# Humanized Bone Model Identifies BMP6 as a Multifunctional Regulator in Myeloma Bone Disease

**DOI:** 10.3390/biom15121747

**Published:** 2025-12-18

**Authors:** Jiaxian Wang, Thomas Baardemans, Ricardo de Matos Simoes, Willy Noort, Ruud W. J. Ruiter, Henk-Jan Prins, Susan E. van Hal-van Veen, Huipin Yuan, Joost D. de Bruijn, Anton C. M. Martens, Constantine S. Mitsiades, Sonja Zweegman, Maria Themeli, Richard W. J. Groen

**Affiliations:** 1 Department of Hematology, Amsterdam UMC, VU University Medical Center, Cancer Center Amsterdam, 1081 HV Amsterdam, The Netherlands; 2 Department of Medical Oncology, Dana-Farber Cancer Institute, Harvard Medical School, Boston, MA 02215, USA; 3 Kuros Biosciences BV, 3723 MB Bilthoven, The Netherlands

**Keywords:** multiple myeloma, bone disease, xenograft, osteoblast, BMP

## Abstract

Multiple myeloma (MM) is a plasma cell malignancy that disrupts bone homeostasis by suppressing osteogenesis and promoting osteoclast activity. While most therapeutic interventions to date have focused on targeting tumor cells and reducing osteolysis, we investigate whether osteoinductive strategies can restore bone formation and counteract disease progression. Using a human bone marrow-like scaffold model that enables direct in vivo evaluation of tumor–stroma interactions and human bone formation, we demonstrate that MM-derived mesenchymal stromal cells (MSCs) retain osteogenic potential but are functionally suppressed by MM cells. Transcriptomic profiling of MM-primed MSCs revealed the downregulation of small leucine-rich proteoglycans (SLRPs), *ASPN*, *OGN*, and *OMD*, key mediators of bone morphogenetic protein (BMP) signaling, which governs osteoblast differentiation. Among the BMPs analyzed, BMP6 emerged as a potent inducer of osteogenesis and regulator of the expression of these SLRPs. Notably, BMP6 selectively promoted bone formation without enhancing osteoclastogenesis and attenuated inflammatory and tumor-supportive MSC phenotypes. BMP6 also directly inhibited MM cell proliferation and suppressed IL6-induced growth. These findings highlight BMP6 as a distinct multifunctional regulator warranting further investigation as a potential therapeutic approach, while establishing the humanized model as a valuable platform for dissecting tumor–bone interactions in MM.

## 1. Introduction

Multiple myeloma (MM) is an incurable hematologic malignancy characterized by a clonal expansion of plasma cells within the bone marrow (BM), with severe bone destruction being one of the major complications. At diagnosis, approximately 80% of MM patients present with bone lesions, manifesting as bone pain, fractures, spinal cord compression, and hypercalcemia [1,2]. Under physiological conditions, bone homeostasis is maintained through a tightly regulated balance between osteoblast-mediated bone formation and osteoclast-driven bone resorption. In MM, this equilibrium is markedly disrupted as a result of complex interactions between malignant plasma cells and the BM microenvironment promoting osteoclastogenesis, while concurrently suppressing osteoblast differentiation and function [3,4]. Despite advances in the therapeutic opportunities targeting malignant plasma cells, treatment options for myeloma bone disease (MBD) remain limited. Bisphosphonates, which target osteoclasts, are the current standard of care and can reduce skeletal-related events [5,6,7]. Denosumab, an anti-RANKL monoclonal antibody inhibiting osteoclast formation and activation, has also shown promise in mitigating bone disease [8]. However, these treatments primarily prevent further bone loss and do not promote regeneration. Notably, long-term bisphosphonates administration may even suppress new bone formation [9]. Thus, given that MBD significantly compromises patients’ quality of life and is an increasing cause of morbidity and mortality, there is a substantial unmet clinical need for novel therapeutic strategies that not only inhibit bone resorption but also promote osteogenesis. Agents such as romosozumab, a monoclonal antibody targeting sclerostin that stimulates bone formation, have shown promising results for osteoporosis and are currently being explored for their potential in myeloma-related bone disease.

Osteoblasts, the cells that drive osteogenesis, originate from mesenchymal stromal cells (MSCs) residing in the BM [10]. Over the last two decades, it has become evident that interactions between malignant plasma cells and MSCs play a pivotal role in MM progression and therapy response [11]. While these interactions induce plasma cell proliferation and survival, they also profoundly alter MSC biology. Transcriptional analysis of MSCs from MM patients versus healthy donors has identified deregulated expression of MM survival factors such as *IL-6*, *LIF*, and the WNT signaling antagonist *DKK1* [12,13,14,15,16]. These alterations persist even after successful antitumor treatment [12,17], and have been associated with MSC senescence [18,19,20], bone marrow inflammation [12,21], and potential priming towards adipogenesis [21].

Despite these transcriptional changes, the mechanisms underlying the persistent lack of bone formation and repair, even after successful treatment of malignant plasma cells, remain incompletely understood. Reports on the functional characteristics of MM-derived MSCs have been heterogeneous. Some studies describe reduced proliferative capacity and/or impaired osteogenic differentiation [14,15,18,22], while others report preserved osteogenic potential and even enhanced proliferation [13,23,24,25]. These discrepancies may, in part, reflect differences in culture conditions, particularly the use of fetal bovine serum (FBS), which, unlike platelet lysate (PL), is known to affect MSC functionality and alter the balance of stromal subpopulations in culture [26,27]. Moreover, data from murine models should be interpreted with caution, as species-specific differences may limit their translational relevance to human disease [28,29,30].

To overcome the current limitations in understanding and treating myeloma bone disease, we leveraged a humanized bone marrow scaffold (huBMsc) model [31] that enables the in vivo evaluation of human bone formation and its modulation by malignant plasma cells. Our findings reveal that MM-derived MSCs retain bone-forming potential in vivo, but are functionally suppressed by MM cells. By integrating transcriptomic data with osteogenic differentiation profiles, we identified downregulation of the SLRPs *ASPN*, *OGN*, and *OMD* as mediators of this suppression, pointing to impaired BMP signaling. Among the BMPs tested, BMP6 emerged as a potent regulator that is capable of restoring bone formation, inhibiting RANKL-induced osteoclastogenesis, and attenuating the tumor-supportive phenotype of MSCs, supporting its further exploration as a potential therapeutic strategy for multiple myeloma and its associated bone disease.

## 2. Materials and Methods

### 2.1. Cell Culture, Osteogenic Differentiation, and Coculture

Luciferase (Luc)-transduced L363 cells (L363-Luc) were cultured in RPMI medium (Gibco, Life technologies, Paisley, UK) supplemented with 10% FetalClone™ I serum (GE Healthcare Life Sciences, Marlborough, MA, USA) and 1% streptomycin/penicillin (Gibco, Life technologies, Paisley, UK). Luc-transduced LME1 cells (LME1-Luc) were cultured in IMDM medium (Gibco, Life technologies, Paisley, UK) supplemented with 10% FetalClone™ I serum, 1% streptomycin/penicillin, 20 µg/mL transferrin (Sigma, St. Louis, MO, USA), and 50 µM β-mercaptoethanol (Life technologies, Grand Island, NE, USA). HS5 and HS27A cell lines were cultured in DMEM (Gibco, Life technologies, Paisley, UK) supplemented with 10% FetalClone™ I serum, and 1% streptomycin/penicillin. Authenticity of the cell lines was verified by STR profiling (GenePrint 10 System Promega, Madison, WI, USA). Primary MSCs (pMSCs) were cultured in α-MEM medium (Gibco, Life technologies, Paisley, UK) supplemented with 10% human platelet lysate, 1% streptomycin/penicillin, and 1% heparin (LEO Pharma B.V, Amsterdam, The Netherlands), with a maximum of 3 passages.

Mono- and cocultures were performed as follows: 10,000 of L363-Luc or LME1-Luc cells were plated in a white opaque 96-well flat-bottom plate in the absence or presence of BMP2 or BMP6 (both R&D systems, Bio-Techne Ltd, Abingdon, UK) in a range of 10–150 ng/mL. After 48 h, MM cell growth was quantified with bioluminescent imaging (BLI) 30 min after the addition of beetle luciferin (100 μg/mL; Promega, Madison, WI, USA), using a GloMax^®^ Discover System (Promega, Madison, WI, USA). For primary MM cells, the mononuclear bone marrow cells were seeded at a density of 10,000 cells/well in a 96-well flat-bottom plate. The cell number of primary MM cells identified by double positive staining for CD138 (clone 281-2, Biolegend, San Diego, CA, USA) and CD38 (clone HIT2, Biolegend, San Diego, CA, USA) was determined by flow cytometry, using beads (Beckman Coulter, Beckman Coulter, Brea, CA, USA).

Coculture experiments were initiated with the seeding of pMSCs or the stromal cell lines HS5 or HS27A at a density of 10,000 cells/well in a white opaque 96-well flat-bottom plate. After o/n incubation, 5000 L363-Luc or LME1-Luc cells were added in the absence or presence of BMP2 or BMP6 at a final concentration of 150 ng/mL. MM cell growth was measured 48 h later, using BLI as described above. To assess the indirect effects of BMP2 and BMP6, stromal cells (pMSCs, HS5 and HS27A) were plated at a density of 2500 cells/well in a white opaque 96-well flat-bottom plate and incubated with BMP2 or BMP6 (150 ng/mL) in the presence of 0.2 mM L-ascorbic acid phosphate. After four (HS5 and HS27A) or eight (pMSCs) days, BMPs were washed off with PBS, followed by the addition of 5000 of L363-Luc or LME1-Luc cells. MM cell growth was measured 48 h later, using BLI as described above.

In vitro osteogenic differentiation of pMSCs was performed in osteogenic medium, comprising α-MEM medium supplemented with 10% FetalClone™ I serum, 1% streptomycin/penicillin, 0.2 mM L-ascorbic acid phosphate (Sigma, St. Louis, MO, USA), and 10 nM dexamethasone (Sigma, St. Louis, MO, USA). For transcriptional profiling, 200,000 pMSCs were loaded onto scaffolds consisting of three particles made of biphasic calcium phosphate (BCP, Kuros Biosciences, Bilthoven, The Netherlands) and β-tricalcium phosphate (β-TCP; Kuros Biosciences, Bilthoven, The Netherlands or ChronOS, DePuy Synthes, West Chester, PA, USA), and cells were collected after 8h, 24h, 3 days, and 7 days of differentiation and stored in Trizol reagent until further processing. For coculture with MM cells, 10,000 pMSCs were loaded to a disk-shape β-TCP scaffold in white opaque, 96-well flat-bottom plates and osteogenically differentiated. Ten days post-differentiation towards osteoblasts, 10,000 L363-Luc or LME1-Luc cells were added for coculture. Then, 48 h after the initiation of the coculture, MM cell growth was quantified by BLI, as described above.

All primary samples were obtained with informed consent and approval by the institutional medical ethical committee.

### 2.2. Bone Evaluation in Human BM-like Scaffold (huBMsc) Mice

Scaffolds containing human bone were created as described previously [31]. In brief, 3 hybrid scaffolds consisting of three 2 to 3 mm β-tricalcium phosphate (β-TCP) particles loaded with 200,000 human MSCs were cultured under osteogenic medium for one week and subsequently implanted subcutaneously into RAG2^−/−^γ_c_^−/−^ mice. Six weeks after implantation, mice were sacrificed and scaffolds were harvested and fixed in formalin, followed by processing and embedding in paraffin. Bone formation was analyzed using Hematoxylin and Eosin (HE) staining. Images (*n* = 3) were analyzed and scored with 0 for no bone formation, 1 for bone formation in 1 particle, 2 for bone formation in >1 particle, or 3 for extensive bone formation in 3 particles.

To study the direct impact of MM cells on bone formation in vivo, after in vitro osteogenic differentiation, and prior to implantation of the scaffolds into mice, scaffolds were incubated 6 h with patient-derived MM cells or cells of the MM cell line LME-1 to allow for adherence and infiltration of the MM cells. Tumor growth was analyzed by bioluminescent imaging, as described previously [31]. Six weeks after implantation, mice were sacrificed and scaffolds were harvested. Scaffolds were processed for bone evaluation as described previously [26], or minced and cultured in pMSC medium to retrieve and expand stromal cells from the scaffolds. When a confluent layer of MSCs was achieved, MSCs were harvested and analyzed by flow cytometry and a pellet was snap-frozen in liquid nitrogen until further processing.

### 2.3. Gene Expression Analysis by the Affymetrix GeneChip System or qRT-PCR

Total RNA was isolated using Trizol extraction (Invitrogen, Carlsbad, CA, USA), according to the manufacturer’s instructions, and concentrated using the RNeasy MinElute Cleanup Kit (Qiagen, Hilden, Germany). For microarray analysis, using Human Gene 1.0 ST Chips, a minimum of 2 μg RNA was sent to the DFCI Microarray Core Facility. For qRT-PCR, 500 ng–1 μg RNA was used for reverse transcription with the Superscript IV kit (Invitrogen, Carlsbad, CA, USA). Quantitative PCR was performed with SYBR green reagent (Roche, Basel, Switzerland) with a LightCycler machine (Roche, Basel, Switzerland). The relative gene expression levels were calculated with the 2ΔΔCt method and normalized to *GAPDH*. The primer sequences are listed in Table S1.

### 2.4. Bioinformatics Analysis

Differential expression analysis (DEA) was performed in R (v4.5.0), using Limma (v3.64.1) [32] for microarray datasets and DESeq2 (v1.48.1) [33] for RNA-seq datasets.

Differentially expressed genes (DEG, *p*-adjusted <0.05 and fold change > 1.5) overlapping between in vivo MM primed (Supplementary Figure S1A), in vitro scaffold (Supplementary Figure S1B), and in vitro plastic (GSE129036: [34]) datasets were filtered for inverse correlation and z-scores were plotted using ComplexHeatmap(v2.24.0) [35]. Inversely correlated genes were compared with DEGs from AML (GSE92778: [36]), ALL (GSE101425; [37]), and MM (GSE80608: [16]) datasets to obtain MM-specific genes.

### 2.5. Alkaline Phosphatase Staining

Primary MSCs were seeded in a 12-well plate at a density of 50,000 cells/well in α-MEM medium supplemented with 10% FBS. The next day, cells were cultured with BMP2 or BMP6 (150 ng/mL) in the presence or absence of 0.2 mM L-ascorbic acid phosphate. Eight days after incubation, the alkaline phosphatase activity was detected with the BCIP/NBT kit (Sigma, St. Louis, MO, USA), according to the manufacturer’s instructions. Briefly, cells were fixed in cold methanol for 5 min, followed by washing with PBS. Subsequently, cells were stained with the BCIP/NBT solution for 20 min at room temperature in the dark. After staining, cells were washed with PBS and photographed.

### 2.6. Osteoclast Differentiation

Peripheral blood mononuclear cells (PBMCs) from buffy coats of healthy donors (Sanquin blood-bank) were first isolated by Ficoll-Paque (Cytiva, Uppsala, Sweden) density centrifugation. Then, CD14+ monocytes were positively enriched by EasySep™ magnetic sorting (Stemcell Technologies, Vancouver, BC, Canada), followed by seeding at a density of 300,000 cells/cm^2^ in tissue culture-treated plates (Greiner Bio-One, Frickenhausen, Germany). After culturing in α-MEM medium supplemented with 10% FBS and M-CSF (25 ng/mL, Peprotech, New Jersey, NJ, USA) for 3 days, the media were changed into the different conditions tested, as indicated in the figure, and refreshed every 3–4 days. Two weeks after differentiation, the osteoclast differentiation was evaluated with tartrate-resistant acid phosphatase (TRAP) staining, as previously described [38]. In brief, cells were first washed with PBS and fixed in 4% PFA at room temperature for 15–30 min. After fixation, the TRAP activity was determined by using tartaric acid buffer (Sigma, St. Louis, MO, USA) containing naphthol AS-BI phosphate (Sigma, St. Louis, MO, USA) and Fast Red TR salt (Sigma, St. Louis, MO, USA) as the substrate. The TRAP-positive multinucleated cells containing three or more nuclei were considered to be osteoclasts and the number was enumerated by counting five random fields in different areas of each well under an inverted microscopy (Leica, Amsterdam, The Netherlands) for a total of two independent experiments.

### 2.7. Statistical Analysis

The data were plotted using GraphPad Prism 9.5.1. The *p* value was calculated based on the unpaired, two-tailed *t*-test. The illustration of the data was described in detail in the legends.

## 3. Results

### 3.1. Evaluation of In Vivo Bone Formation by MM-Derived MSCs and Direct Inhibition by MM Cells Using the huBMsc Model

**Figure 1:**
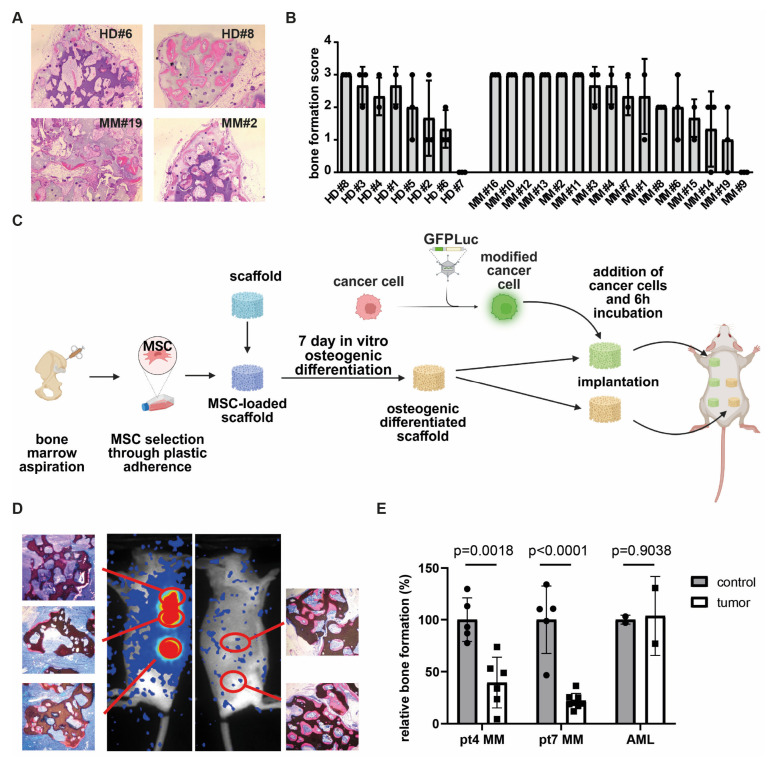
MM cells inhibit osteogenic differentiation in huBMsc model. (**A**) In vivo bone formation of healthy donor and MM-patient-derived MSCs, as visualized by H&E staining. Donors shown are representative of good (HD #8 and MM #2) and poor (HD #6 and MM #19) bone formation. (**B**) Quantification of the in vivo bone formation capacity of MSCs, either from healthy donors or myeloma patients. (**C**) Schematic outline of in vivo priming of healthy MSCs by MM in the huBMsc model. Created in BioRender. Themeli, M. (2025) https://BioRender.com/h7nz41g. (**D**) Representative images showing the in vivo bone formation (HE staining) and tumor growth (bioluminescent imaging) in mice implanted with healthy donor MSCs coated scaffolds alone (right) or coated with MSCs and MM cells (left). (**E**) Quantification of the in vivo bone formation by healthy donor MSCs in the presence of MM or AML cells. *p* value was determined by a two-way unpaired student *t* test.

To determine whether MM patients could benefit from osteoinductive therapy aimed at restoring bone formation, we first assessed whether MM-derived MSCs retain the capacity to form a bone matrix in vivo. MSCs were culture-expanded, using PL as a substitute for FBS [26] from bone marrow aspirates of sixteen newly diagnosed MM patients and eight age-matched healthy donors undergoing hip replacement surgery. Passage 3 MSCs were seeded onto β-TCP scaffolds (*n* = three per donor), and following one week of osteogenic induction, the scaffolds were implanted subcutaneously into immunodeficient RAG2^−/−^γ_c_^−/−^ knockout mice. After six weeks of in vivo development, the mice were sacrificed and the scaffolds were harvested for histological evaluation. Hematoxylin and eosin (H&E) staining revealed that MSCs derived from the majority of both healthy donors (seven out of eight) and MM patients (15 out of 16) were capable of forming bone in vivo (Figure 1A,B). The extent of bone formation was heterogenous, ranging from a bone formation score from one to three, and was comparable between MSCs derived from healthy donors and those from MM patients (Figure 1B, *p*-value 0.3945). These findings demonstrate that MSCs obtained from MM-involved bone marrow at diagnosis retain osteogenic potential. This suggests that the transcriptional and compositional alterations previously reported in MM-derived MSCs are not permanently fixed and may be reversible at this stage of the disease.

To further investigate the mechanisms underlying the suppression of osteogenesis in MM, we conducted co-implantation studies, using our huBMsc model. In this modified approach, non-mineralized scaffolds loaded with healthy donor-derived MSCs were bilaterally implanted into mice: the left flank received scaffolds co-implanted with MM cells, while the right flank served as a control, receiving MSC-loaded scaffolds without tumor cells (Figure 1C). Unlike our earlier design, where MM cells were introduced into pre-mineralized constructs [31], this setup enabled us to assess whether MM cells directly inhibit de novo bone formation in vivo. Using this system, we observed reduced bone formation in scaffolds that were retrieved from sites co-implanted with MM cells, as confirmed by bioluminescent imaging (Figure 1D). Quantification revealed a consistent inhibitory effect across both primary MM donors investigated. Notably, this inhibition was not observed when acute myeloid leukemia (AML) cells were used as a tumor control (Figure 1E), indicating that the suppression of osteogenesis is specific to MM and not merely due to spatial competition. Collectively, these data indicate that MM cells suppress osteogenic differentiation through a reversible mechanism, rather than by inducing permanent changes in MSCs.

### 3.2. Identification of Small Leucine-Rich Proteoglycans ASPN, OGN, and OMD as Intermediates of Bone Formation Inhibited in MM

**Figure 2:**
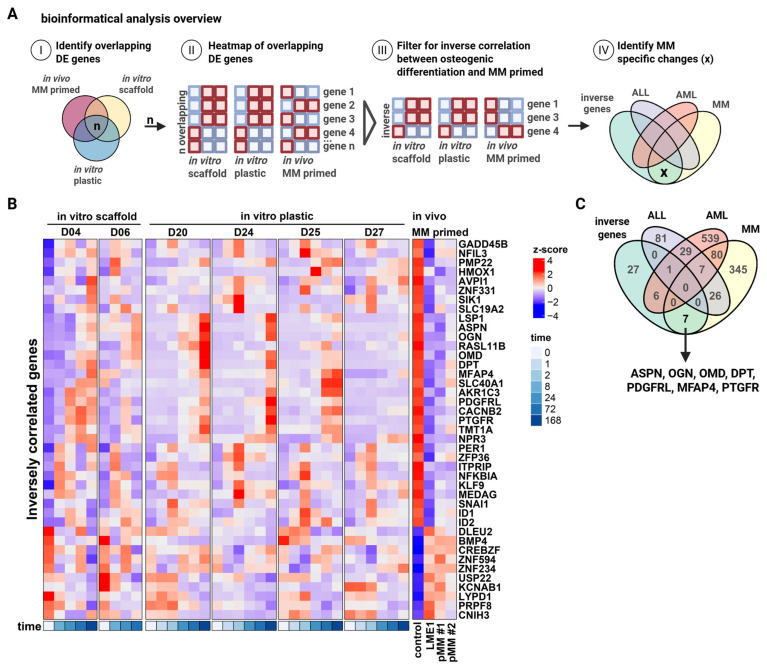
Transcriptomic profiling of “MM-primed” MSCs reveals the small leucine-rich proteoglycans, *ASPN*, *OGN*, and *OMD*, as being potential mediators of MM bone disease. (**A**) Overview of the bioinformatical analysis workflow. (**B**) Heatmap showing the differentially expressed genes that are inversely correlated between in vitro osteogenic differentiation (BCP scaffold and plastic) and in vivo primed MSCs by MM. (**C**) Venn diagram showing the overlapping between our inversely correlated genes dataset and publicly available datasets comparing the transcriptomics of ALL, AML, and MM patient and healthy donor-derived MSCs. Created in BioRender. Themeli, M. (2025) https://BioRender.com/887ffa3.

To gain insight into how MM cells suppress osteogenesis, we performed transcriptomic analysis of MSCs retrieved from scaffolds exposed to MM cells (referred to as MM-primed MSCs) and from control scaffolds. After scaffold retrieval from the mice, MSCs were isolated by plastic adherence, expanded ex vivo in PL-supplemented medium, and subjected to gene expression profiling using the Affymetrix platform (Supplementary Figure S1A). Human origin of the MSCs was confirmed by CD73 and CD90 expression and lack of human and murine CD45. In parallel, transcriptional profiles were generated from healthy donor-derived MSCs undergoing osteogenic differentiation over time on osteoinductive scaffolds (Supplementary Figure S1B). To identify genes involved in MM-mediated inhibition of osteogenesis, we compared the MM-primed MSC dataset with both our osteogenic differentiation dataset and a third publicly available RNA-sequencing time-course of MSC osteogenesis (GSE129036; [34]) (Figure 2A(I,II)). This comparison resulted in 74 transcripts that were consistently represented across all three datasets (Supplementary Figure S1C,D). Among these, we focused on genes showing inverse regulation (Figure 2A(III)), downregulated in MM-primed MSCs but upregulated during osteogenic differentiation, resulting in a subset of 41 transcripts associated with osteogenesis but suppressed by MM cells (Figure 2B and Supplementary Figure S1E). To assess clinical relevance and specificity to MM, we next compared these 41 genes to transcriptomic datasets of MSCs derived from patients with AML (GSE101425; [38]), ALL (GSE92778; [37]), and MM (GSE80608; [16]) (Figure 2A(IV)). This analysis identified seven transcripts selectively altered in response to MM-priming, but not in AML- or ALL-affected MSCs, suggesting MM-specific regulation (Figure 2C). Notably, three of these seven genes, *ASPN*, *OGN*, and *OMD*, belong to the small leucine-rich proteoglycan (SLRP) family, which has been implicated in extracellular matrix organization and bone formation.

### 3.3. Bone Morphogenetic Proteins Regulate Small Leucine-Rich Proteoglycans During Osteogenic Differentiation

**Figure 3:**
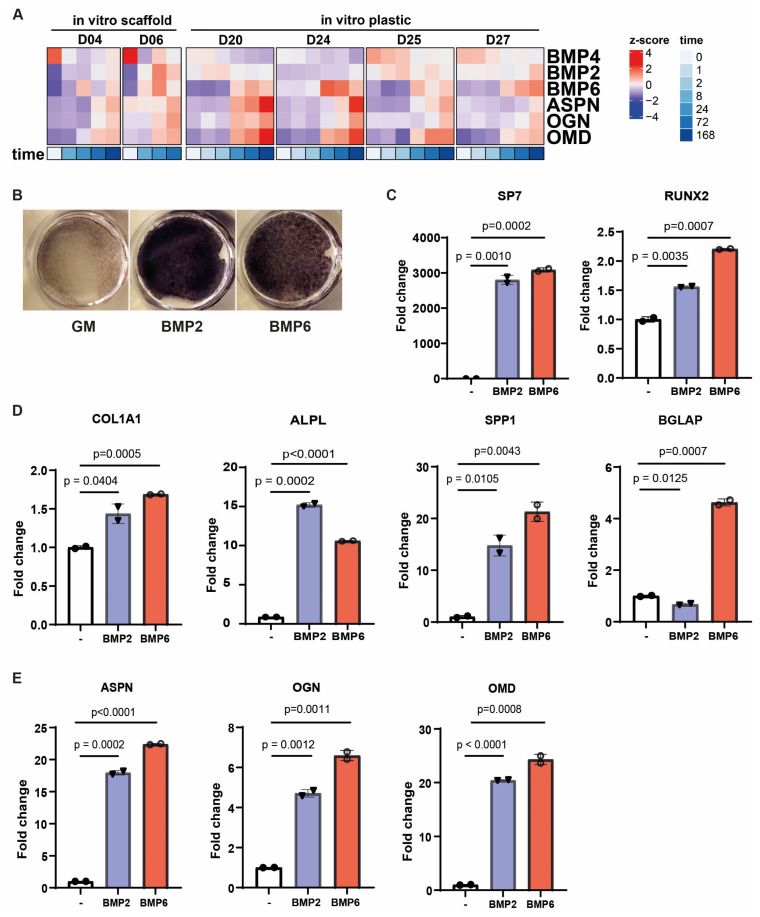
BMPs can regulate small leucin rich proteoglycans *ASPN*, *OGN*, and *OMD* during osteogenic differentiation. (**A**) Heatmap illustrating how the expression of *BMP2*, *BMP4*, and *BMP6* aligns with three *ASPN*, *OGN*, and *OMD* during in vitro osteogenic differentiation (BCP scaffold and plastic). (**B**–**E**) MSCs were treated with BMP2 or BMP6 in the presence of L-ascorbic acid phosphate for 8 days and (**B**) stained for alkaline phosphatase activity as a measure of osteogenic differentiation, as well as being analyzed by qRT-PCR for the expression of (**C**) osteogenic transcription factors, (**D**) classical markers of osteogenesis, and (**E**) the three identified SLRP genes *ASPN*, *OGN*, and *OMD*. Data are shown as mean ± sd and are representative of three independent experiments. Statistical significance was determined by a two-way unpaired *t* test.

As bone morphogenetic proteins (BMPs) have been implicated in the regulation of the small leucine-rich proteoglycans asporin (ASPN), osteoglycin (OGN), and osteomodulin (OMD) [39], we analyzed the BMP expression profiles in our scaffold-based in vitro osteogenic differentiation dataset and in the culture plastic-based dataset from Lutsik et al. [34]. *BMP2*, *BMP4*, and *BMP6* showed differential expression over time during osteogenic differentiation in both datasets (Figure 3A and Supplementary Figure S1F). Interestingly, whereas *BMP4* was downregulated, *BMP2* and *BMP6* were upregulated, showing expression patterns that aligned with *ASPN*, *OGN*, and *OMD*. Next, we evaluated the osteoinductive potential of BMP2 and BMP6 and their ability to modulate SLRP expression in primary MSCs. Alkaline phosphatase activity confirmed that both BMPs promoted osteogenic differentiation in vitro (Figure 3B). This was further supported by qRT-PCR, which showed increased expression of the osteogenic transcription factor *SP7*, and, to a lesser extent, *RUNX2* (Figure 3C), along with classical markers including *COL1A1*, *ALPL*, *SPP1*, and *BGLAP* (Figure 3D). Of note, the induction of *BGLAP*, a marker associated with more mature osteoblasts, was observed only in response to BMP6. Moreover, BMP6, as well as BMP2, increased the expression of *ASPN*, *OGN*, and *OMD*, suggesting that these proteoglycans may indeed act as downstream targets of BMP signaling (Figure 3E).

### 3.4. BMP2 and BMP6 Abolish the Supportive Role of MSCs in the MM Microenvironment

**Figure 4:**
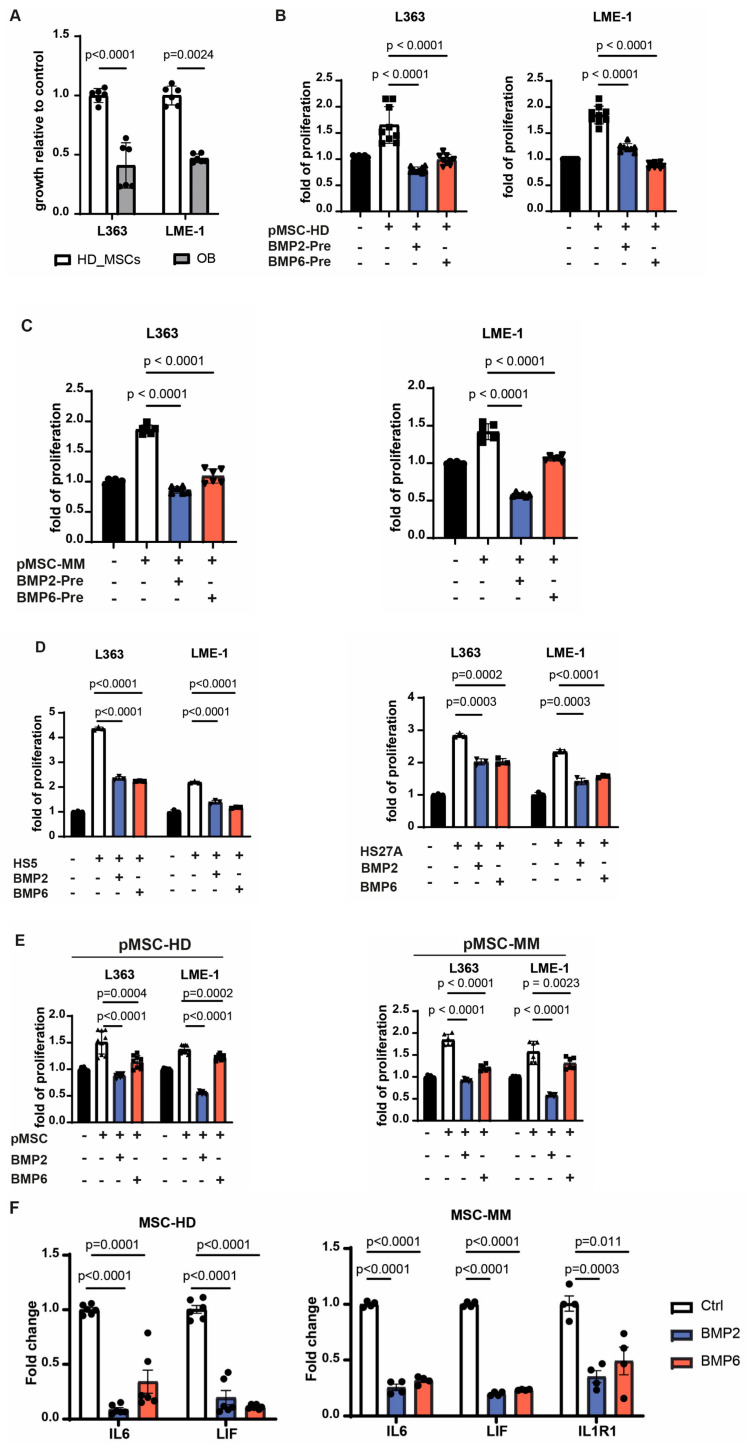
BMP2 and BMP6 can abolish the supportive role of MSCs in the microenvironment. (**A**) Primary MSCs were induced towards osteoblasts for one week, followed by coculture with MM cells two days later. The error bar represents mean ± sd of three replicates from two independent experiments. (**B**,**C**) Primary MSCs from healthy donors (**B**) or MM patients (**C**) were incubated with BMP2 or BMP6 in the presence of L-ascorbic acid phosphate for 8 days. After removal of BMPs, MM cells were added and cocultured for two days. Shown data are mean ± sd pooled from three healthy donors or two patients with three replicates each. (**D**) MM cells were cultured with BMP2 or BMP6 in the presence of HS5 cells or HS27A cells for two days. Data present as mean ± sd from three independent experiments. (**E**) MM cells were cultured with BMP2 or BMP6 in the presence of primary MSCs from healthy donors or MM patients for two days. Data are mean ± sd pooled from three healthy donors or two patients with three replicates each. (**F**) The expression levels of the inflammatory cytokines of primary MSCs from either MM patients or healthy donors after incubation with BMP2 or BMP6 in the presence of vitamin C for 8 days were evaluated by qRT-PCR. Data are shown as mean ± sd pooled from three donors or two patients with duplicates each. Growth of MM cells was assessed by bioluminescent imaging. All the statistical analysis was performed with a two-way unpaired *t* test.

In view of previously reported stroma-induced MM growth and therapy resistance, we next investigated whether inducing osteogenic differentiation in MSCs could attenuate these pro-tumorigenic effects. Indeed, MSCs that differentiated toward the osteoblastic lineage lost their ability to promote MM cell growth (Figure 4A). Similarly, pre-treatment of MSCs from both healthy donors and MM patients with BMP2 or BMP6 abolished their supportive capacity (Figure 4B,C). Since stromal pre-conditioning is not feasible in clinical settings, we also assessed the effects of BMP2 and BMP6 by adding them directly to co-cultures of stromal and MM cells. Initial experiments using the stromal cell lines HS5 and HS27A, characterized by high expression of IL6 and CXCL12, respectively [40,41], showed that BMP2 and BMP6 reduced their ability to support MM cell growth (Figure 4D). This inhibitory effect was even more pronounced in co-cultures with primary MSCs from healthy donors and MM patients (Figure 4E). Recent studies suggest that stromal inflammation, marked by elevated *IL6*, *LIF*, and *IL1R1* expression, contributes to MM progression in the BM microenvironment [12]. Consistent with this, qPCR analysis showed increased expression of these genes in MSCs from MM patients compared to healthy donor-derived MSCs, with *IL1R1* being undetectable in the latter (Figure 4F). Notably, treatment of stromal cells with BMP2 or BMP6 reduced the expression levels of all three inflammatory mediators. These findings suggest that BMP2 and BMP6 not only promote osteogenesis but also counteract stromal-mediated myeloma growth by downregulating pro-inflammatory cytokine expression, including IL6.

### 3.5. BMP2 and BMP6 Inhibits MM Growth, as Well as IL6-Induced Proliferation

**Figure 5:**
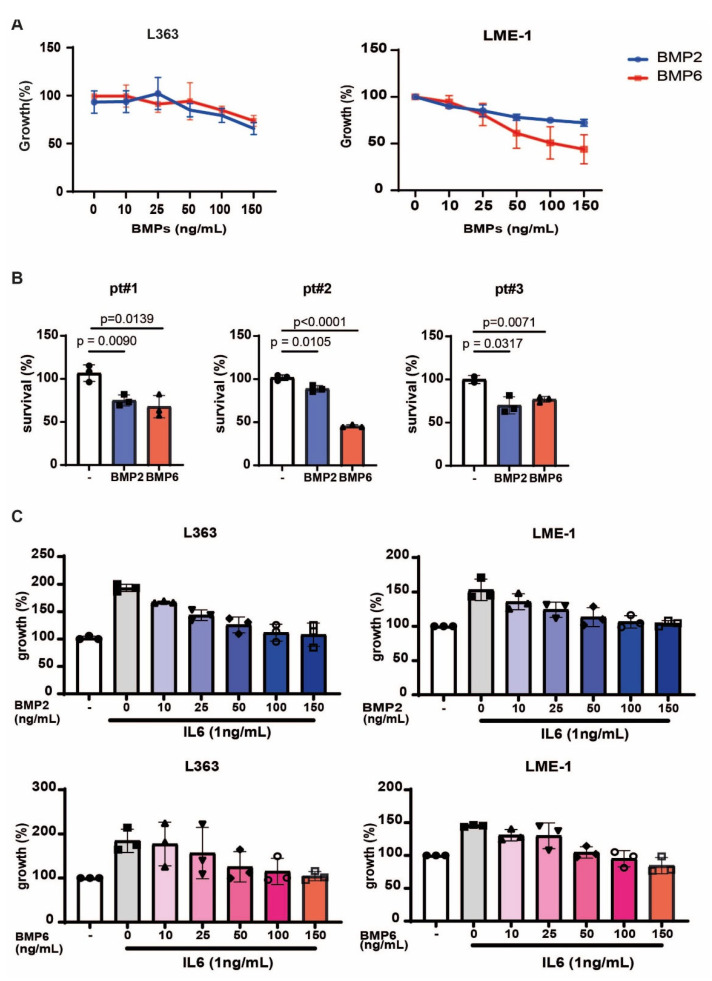
BMP2 and BMP6 inhibit MM growth and interfere with IL6-induced proliferation. (**A**) Growth curves of MM cells treated with increasing concentrations (0–150 ng/mL) of BMP2 or BMP6. Data are presented as mean ± sd from three independent experiments. (**B**) Survival of primary MM cells from three MM patient-derived BM samples incubated with BMP2 or BMP6 (150 ng/mL). Data are presented as mean ± sd from triplicates. Statistical analysis was performed with two-way unpaired *t* test. (**C**) Growth of MM cells treated with increasing concentrations (0–150 ng/mL) of BMP2 or BMP6 in the presence of 1 ng/mL IL6. Data are presented as mean ± sd from three independent experiments.

In addition to the indirect effect via the microenvironment, we also investigated the direct anti-myeloma potential of BMP2 and BMP6. In line with previous reports [42,43,44,45,46], we confirmed that both BMPs inhibited the growth of the MM cell lines L363 and LME-1, and reduced the viability of primary malignant plasma cells from three MM-derived BM samples (Figure 5A,B). Given the key role of IL6 in supporting MM survival and proliferation [47,48], and our observation that BMP2 and BMP6 reduced IL6 expression in MSCs, we next assessed whether they could directly counteract IL6-induced myeloma growth. As shown in Figure 5C, IL6 stimulated the proliferation of L363 and LME-1 cells, which was suppressed to basal levels by increasing concentrations of BMP2 and BMP6. Thus, in addition to osteogenic differentiation and disrupting stromal support for MM, BMP2 and BMP6 exert direct anti-myeloma effects and inhibit IL6-mediated proliferation.

### 3.6. BMP6, but Not BMP2, Inhibits the RANKL-Dependent Osteoclastogenesis

**Figure 6:**
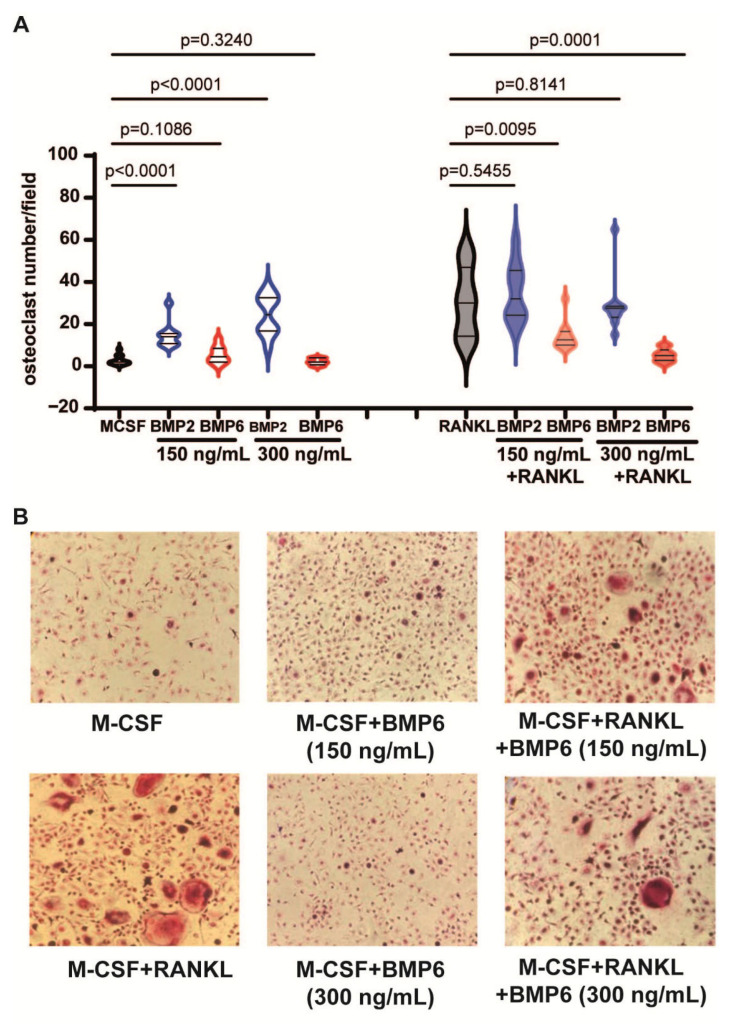
BMP6, but not BMP2, inhibits RANKL-dependent osteoclastogenesis. (**A**) Violin plots showing the numbers of osteoclasts per field differentiated from monocytes following treatment with BMP2 and BMP6 alone or in the presence of RANKL. Data shown are pooled data from two independent experiments. (**B**) Representative images of monocytes treated with RANKL, BMP6 or the combination. All the statistical analysis was performed with a two-way unpaired *t* test.

Finally, as osteoclasts play a critical role in myeloma bone disease, we evaluated the effects of BMP2 and BMP6 on osteoclast formation. CD14+ monocytes were cultured with BMP2 or BMP6 for two weeks in the presence or absence of RANKL. Tartrate-resistant acid phosphatase (TRAP) staining revealed that BMP2 enhanced osteoclast formation but did not affect RANKL-induced osteoclastogenesis (Figure 6A). In contrast, BMP6 had no osteoclastogenic effect and inhibited RANKL-induced osteoclast formation in a dose-dependent manner (Figure 6A,B). Collectively, these data show that unlike BMP2, BMP6 does not induce osteoclast formation and can inhibit RANKL-dependent osteoclastogenesis.

## 4. Discussion

In this study, we used our humanized bone marrow scaffold (huBMsc) model to demonstrate that MM-derived MSCs retain their intrinsic capacity for in vivo bone formation, while malignant plasma cells actively suppress osteogenesis with healthy donor-derived MSCs. Transcriptomic profiling indicated reduced BMP signaling, with the extracellular matrix proteins *ASPN*, *OGN*, and *OMD*, members of the small leucine-rich proteoglycan (SLRP) family, emerging as key intermediates. A functional comparison of BMP2 and BMP6 revealed that both promote osteogenic differentiation, abrogate the MM-supportive phenotype of MSCs, and inhibit MM cell proliferation directly; notably, only BMP6 additionally suppressed RANKL-induced osteoclastogenesis.

Our observation that MSCs derived from active lesions of MM patients retain their in vivo osteogenic potential in the absence of MM cells is consistent with earlier in vitro studies by Kassen et al. [23], which showed that the frequency of MSC progenitors and their osteogenic differentiation potential remain unaffected in MM patients. Moreover, it also aligns with recent clinical data indicating that the impact of MM on osteogenesis is spatiotemporal, with reduced bone formation localized to active tumor sites, while adjacent regions remain osteogenically competent [49].

Concomitantly, performing transcriptional analysis to gain mechanistic insight into how MM cells suppress osteogenesis, we identified a set of extracellular matrix genes, i.e., *ASPN*, *DPT*, *OGN*, *OMD*, and *MFAP4*, that were consistently downregulated in MM-primed MSCs but upregulated during osteogenic differentiation. Given their previously described role in collagen matrix organization [50,51,52], this expression pattern offers a molecular explanation for the impaired bone formation observed in vivo. Of particular interest were *ASPN*, *OGN*, and *OMD*, members of the small leucine-rich proteoglycan (SLRP) family, which showed coordinated regulation with *BMP2* and *BMP6* in our datasets, in line with earlier reports linking their expression to BMP6 activity [39]. Their functional relevance is underscored by the observation of Sworder et al. [53], who identified these three SLRPs as markers of MSC clones with robust in vivo bone-forming capacity compared to those only forming fibrous tissue. Collectively, these observations suggest that MM-mediated downregulation of SLRPs disrupts BMP-driven osteogenic differentiation and matrix organization, thereby contributing to impaired bone formation in the myeloma niche.

While restoring osteoblast function is essential to counteracting myeloma bone disease, effective treatment must also address the pathological bone resorption driven by osteoclast activation. We found that BMP6, unlike BMP2, does not promote osteoclast formation and instead inhibits RANKL-induced osteoclastogenesis. BMP2 has been shown to increase osteoclast number and activity in preclinical models [54,55], and has been associated with osteolytic effects in some clinical settings [56,57]. For BMP6, limited clinical observations do not indicate enhanced osteoclast activity or bone resorption [58,59]. A likely explanation lies in receptor specificity: BMP2 preferentially signals via BMPR1A, whereas BMP6 exhibits a higher affinity for ACVR1 [60,61,62]. This receptor preference may help explain why the findings of Gooding et al. [63] initially appear contradictory to ours, yet likely reflect a shared mechanism related to differential BMP receptor engagement. In their murine MM model, BMP signaling inhibition improved bone disease via anti-resorptive effects, attributed to blockade of BMPR1A-mediated pathways using a receptor antagonist (LDN193189) or BMPR1A-Fc ligand trap. However, neutralization of BMP6 had no impact on bone mass or tumor growth, suggesting that BMP6–ACVR1 signaling does not drive osteoclastogenesis in this context. Taken together, these findings illustrate how differential receptor usage can shape the divergent roles of BMP ligands in bone remodeling.

Beyond its skeletal effects, MM has been shown to remodel the bone marrow niche by promoting a persistent inflammatory state in MSCs, marked by elevated expression of *IL6*, *LIF*, and *IL1R1* [12,21]. This inflammatory phenotype persists even after successful antitumor therapy and may contribute to the limited long-term efficacy of current treatment regimens. In this context, we identified BMP signaling as a potential strategy to simultaneously suppress MSC-mediated inflammation, attenuate their tumor-supportive phenotype, and restore osteogenic function. Both BMP2 and BMP6 reduced *IL6* expression in MSCs and counteracted IL6-induced MM cell proliferation, in line with previous reports demonstrating BMP-induced suppression of MM growth and survival [45,64]. These findings reinforce the urgent need for therapies that not only limit bone destruction but also restore stromal function: a clinical gap that is not addressed by current anti-resorptive agents. Given its ability to modulate inflammation, promote osteogenesis, and counter stromal support of tumor cells, BMP6 may be particularly relevant for MM patients with active bone disease and persistent stromal dysregulation, despite remission of tumor burden. Future stratification efforts may help identify patients that are most likely to benefit from BMP6-targeted interventions.

Although the huBMsc model offers a unique platform to study human MSC behavior in a myeloma context, it does not recapitulate all components of the disease. In particular, the absence of an adaptive immune system limits our ability to evaluate its role in MM progression and bone remodeling. Nonetheless, myeloid cells and functional osteoclasts are present in the RAG2^−/−^γ_c_^−/−^ mice used here, and previous work has shown that these cells can be activated by MM cells [31]. Finally, our study focused on the stromal compartment and did not explicitly test the ability of BMP6 to counteract other MM-derived inhibitory signals such as HGF, activin A, or DKK1. Future studies should address whether BMP6 can overcome these additional suppressive signals to determine its robustness in the complex MM microenvironment.

## 5. Conclusions

Overall, our findings identify BMP6 as a distinct and promising multi-functional regulator with the capacity to promote bone formation, limit bone resorption, and attenuate stromal support of tumor growth in multiple myeloma, supporting its further investigation as a potential therapeutic approach for myeloma bone disease.

## Data Availability

The raw data supporting the conclusions of this article will be made available by the authors upon request.

## Supplementary Materials

The following supporting information can be downloaded at: https://www.mdpi.com/article/10.3390/biom15121747/s1, Figure S1. Sample collection and supporting transcriptomic data. Tabel S1. Primer sequences.
