# Supplementary material for: Humanized Bone Model Identifies BMP6 as a Multifunctional Regulator in Myeloma Bone Disease
Source: Biomolecules. 2025 Dec 18;15(12):1747. doi: 10.3390/biom15121747 (PMC12731031; doi:10.3390/biom15121747)
Supplement: Supplementary file 1 [file biomolecules-15-01747-s001.zip › biomolecules-3990802-supplementary.pdf]

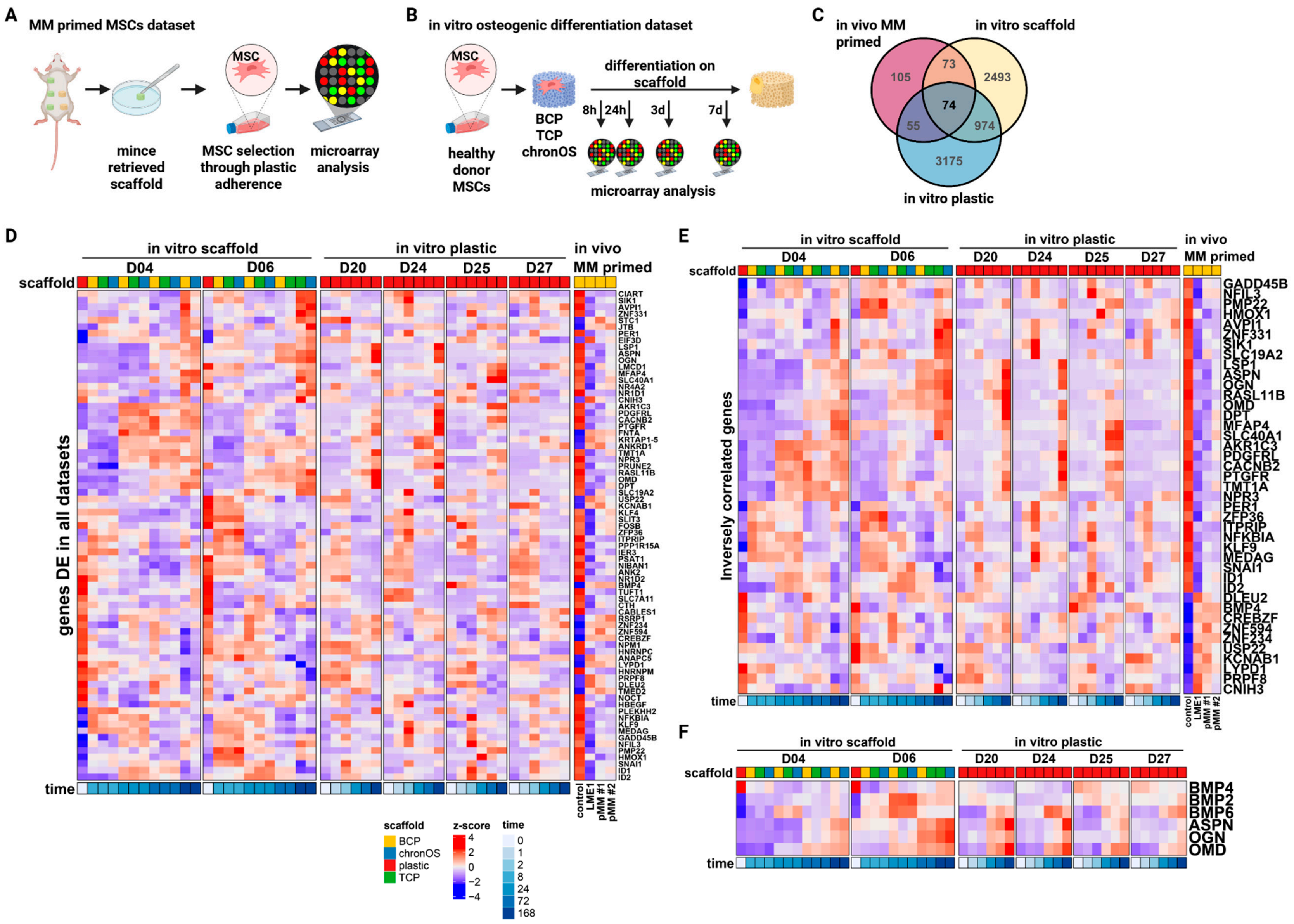
A
MM primed MSCs dataset
mince retrieved scaffold
MSC
MSC selection through plastic adherence
microarray analysis
B
in vitro osteogenic differentiation dataset
healthy donor MSCs
BCP
TCP
chronOS
differentiation on scaffold
8h
24h
3d
7d
microarray analysis
C
in vivo MM primed
in vitro scaffold
in vitro plastic
105
73
2493
74
55
974
3175
D
in vitro scaffold
in vitro plastic
in vivo MM primed
D04
D06
D20
D24
D25
D27
scaffold
genes DE in all datasets
CIART
SIK1
AVPI1
ZNF331
STC1
JTB
PER1
EIF3D
LSP1
ASPN
OGN
LMCD1
MFAP4
SLC40A1
NR4A2
NR1D1
CNIH3
AKR1C3
PDGFRL
CACNB2
PTGFR
FNTA
KRTAP1-5
ANKRD1
TMT1A
NPR3
PRUNE2
RASL11B
OMD
DPT
SLC19A2
USP22
KCNAB1
KLF4
SLIT3
FOSB
ZFP36
ITPRIP
PPP1R15A
IER3
PSAT1
NIBAN1
ANK2
NR1D2
BMP4
TUFT1
SLC7A11
CTH
CABLES1
RSRP1
ZNF234
ZNF594
CREBZF
NPM1
HNRNPC
ANAPC5
LYPD1
HNRNPM
PRPF8
DLEU2
TMED2
NOCT
HBEGF
PLEKHH2
NFKBIA
KLF9
MEDAG
GADD45B
NFIL3
PMP22
HMOX1
SNAI1
ID1
ID2
time
control
LME1
pMM #1
pMM #2
scaffold
BCP
chronOS
plastic
TCP
z-score
4
2
0
−2
−4
time
0
1
2
8
24
72
168
E
in vitro scaffold
in vitro plastic
in vivo MM primed
D04
D06
D20
D24
D25
D27
scaffold
Inversely correlated genes
GADD45B
NFIL3
PMP22
HMOX1
AVPI1
ZNF331
SIK1
SLC19A2
LSP1
ASPN
OGN
RASL11B
OMD
DPT
MFAP4
SLC40A1
AKR1C3
PDGFRL
CACNB2
PTGFR
TMT1A
NPR3
PER1
ZFP36
ITPRIP
NFKBIA
KLF9
MEDAG
SNAI1
ID1
ID2
DLEU2
BMP4
CREBZF
ZNF594
ZNF234
USP22
KCNAB1
LYPD1
PRPF8
CNIH3
time
control
LME1
pMM #1
pMM #2
F
in vitro scaffold
in vitro plastic
D04
D06
D20
D24
D25
D27
scaffold
BMP4
BMP2
BMP6
ASPN
OGN
OMD
time

Supplemental Figure S1. Sample collection and supporting transcriptomic data. (**A**-**B**) Schematic overview of the workflow leading to the collection of transcriptomic data from (**A**) in vivo primed MSCs by MM and (**B**) a time series of MSCs osteogenically differentiated in vitro on ceramic scaffolds (BCP, TCP and chronOS). (**C**) Venn diagram showing the overlap of all differentially expressed genes in MM-primed MSC dataset ("in vivo MM primed") with our osteogenic differentiation dataset ("in vitro scaffold") and a third publicly available RNA-sequencing time-course of MSC osteogenesis ("in vitro plastic"). (**D**) Heatmaps showing the 74 differentially expressed genes that overlap between in vitro osteogenic differentiation on plastic, all scaffold types and in vivo primed MSCs by MM. (**E**) Heatmap showing the inversely correlation between in vitro osteogenic differentiation on plastic, all scaffold types and in vivo primed MSCs by MM. (**F**) Heatmap illustrating how the expression of *BMP2*, *BMP4* and *BMP6* aligns with *ASPN*, *OGN* and *OMD* during in vitro osteogenic differentiation (plastic and all scaffold-types). Created in BioRender. Themeli, M. (2025) https://BioRender.com/4fca5fb.

Tabel S1. Primer sequences.

**gene**	**primer name**	**primer sequence**
GAPDH	qGAPDH_For	TGATGACATCAAGAAGGTGGTGAAG
	qGAPDH_Rev	TCCTTGGAGGCCATGTGGGCCAT
SP7	qSP7_For	TGCTTGAGGAGGAAGTTCAC
	qSP7_Rev	AGGTCACTGCCCACAGAGTA
RUNX2	qRUNX2_For	TCCGGAATGCCTCTGCTGTTATGA
	qRUNX2_Rev	AAGGTGAAACTCTTGCCTCGTCCA
COL1A1	qCOL1A1_For	AGGGCCAAGACGAAGACATC
	qCOL1A1_Rev	AGATCACGTCATCGCACAACA
ALPL	qALPL_For	ACAAGCACTCCCACTTCATCTGGA
	qALPL_Rev	TCACGTTGTTCCTGTTCAGCTCGT
SPP1	qSPP1_For	TCTCCTAGCCCCACAGACC
	qSPP1_Rev	TGGTGAGACTCATCAGACTGG
BGLAP	qBGLAP_For	GGCAGCGAGGTAGTGAAGAG
	qBGLAP_Rev	GATGTGGTCAGCCAACTCGT
ASPN	qASPN_For	CAACAAGAGAGCCAAGAAGCC
	qASPN_Rev	GTTGGTTGGGACTGAGGTCAA
OGN	qOGN_For	GATGAAATGCCCACGTGTCTG
	qOGN_Rev	GCTTGGGAGGAAGAACTGGAA
OMD	qOMD_For	CCCTGTCTGGACACAGAGTTA
	qOMD_Rev	TCATCATCTGGCTCTTGGTCAT
IL6	qIL6_For	AGAGGCACTGGCAGAAAACA
	qIL6_Rev	CAGCTCTGGCTTGTTCCTCA
LIF	qLIF_For	GCCAATGCCCTCTTTATT

	qLIF_Rev	GGAGGTGCCAAGGTACA
IL1R1	qIL1R1_For	GCTGCAGTTGCTGATTCTGG
	qIL1R1_Rev	AATGACTTGTGCGCCCTGTA
